# Angelic Acid Prevents RANKL-Induced Osteoclastogenesis Through Pathway-Biased Inhibition of MAPK–NFATc1 Signaling

**DOI:** 10.3390/cimb48040412

**Published:** 2026-04-17

**Authors:** Lifang Zhang, Mojtaba Tabandeh, Vishwa Deepak

**Affiliations:** 1 Osteoimmunology and Drug Discovery Research Group, Department of Biology, College of Science, Mathematics and Technology, Wenzhou-Kean University, 88 Daxue Road, Wenzhou 325060, China; 2 Dorothy and George Hennings College of Science, Mathematics and Technology, Kean University, 1000 Morris Ave, Union, NJ 07083, USA; 3 Department of Chemistry, College of Science, Mathematics and Technology, Wenzhou-Kean University, 88 Daxue Road, Wenzhou 325060, China; 4 International Frontier Interdisciplinary Research Institute (IFIRI), Wenzhou-Kean University, Wenzhou 325060, China; 5 Wenzhou Municipal Key Laboratory for Applied Biomedical and Biopharmaceutical Informatics, Wenzhou-Kean University, Ouhai, Wenzhou 325060, China; 6 Zhejiang Bioinformatics International Science and Technology Cooperation Center, Wenzhou-Kean University, Ouhai, Wenzhou 325060, China; 7 Zhejiang-Malaysia Joint Laboratory For Rare Medicinal Resources, Wenzhou-Kean University, 88 Daxue Road, Ouhai, Wenzhou 325060, China

**Keywords:** angelic acid, osteoclast, RANKL, MAPK, natural product, bone resorption

## Abstract

Excessive osteoclast activity drives inflammatory bone loss in osteoporosis, rheumatoid arthritis, and periodontitis. Natural compounds represent promising therapeutic candidates with favorable safety profiles; however, few exhibit pathway-biased mechanisms of action. Here, we report that angelic acid (AA), a naturally occurring unsaturated monocarboxylic acid, potently inhibits RANKL-induced osteoclastogenesis. This effect occurs with an IC_50_ of 1.9 µM without cytotoxicity. Mechanistically, AA selectively suppressed RANKL-activated phosphorylation of ERK1/2, p38, and JNK (all three MAPK branches), while leaving NF-κB transcriptional activity unaffected. This preferential MAPK suppression disrupted downstream NFATc1 nuclear translocation, thereby preventing NFATc1-driven transcription of osteoclast-specific effector genes including *TRAP*, *cathepsin K*, and *Atp6v0d2*. These findings identify AA as a novel inhibitor of the RANKL–MAPK–NFATc1 axis, providing a mechanistic foundation for its therapeutic development in osteoporosis and other osteolytic diseases.

## 1. Introduction

Osteoclasts are multinucleated cells derived from monocyte/macrophage lineage that are responsible for bone resorption [1]. While essential for physiological bone remodeling, excessive osteoclast activity leads to pathological bone loss in osteoporosis, rheumatoid arthritis, periodontal disease, and cancer-induced osteolysis [2]. The receptor activator of nuclear factor-κB ligand (RANKL) is the master regulator of osteoclastogenesis, activating multiple signaling cascades including mitogen-activated protein kinases (MAPKs) and nuclear factor-κB (NF-κB), which converge on the master transcription factor NFATc1 [3,4]. Current anti-resorptive therapies including bisphosphonates and denosumab are effective but carry risks of osteonecrosis and atypical fractures with long-term use [5]. Therefore, the identification of alternative therapeutic strategies remains an important goal.

Angelic acid (AA; 2-methylbut-2-enoic acid; C_5_H_8_O_2_; MW = 100.12 g/mol; CAS: 565-63-9) is a naturally occurring α,β-unsaturated monocarboxylic acid found in several plant species, including members of the genus *Angelica* [6]. While *Angelica* species and their preparations have been used traditionally for pain and inflammatory conditions [7], the effects of AA itself on bone metabolism have not been investigated. Notably, extracts from *Angelica* species have been reported to exhibit bone-protective properties [8], suggesting that AA may contribute to these effects. AA has also been associated with anti-inflammatory and analgesic activities, and recent evidence indicates ferroptosis-inducing potential in colorectal cancer cells [9]. The α,β-unsaturated carboxylic acid moiety functions as an electrophilic pharmacophore, enabling covalent modification of nucleophilic protein residues and suggesting a plausible mechanistic basis for its diverse bioactivities [10]. Phytochemically, AA has been reported in the roots and rhizomes of *Angelica archangelica* and related species, including *A. dahurica* and *A. sinensis*, occurring as the free acid or esterified derivatives [6]. Reported isolation approaches for angelic acid-containing fractions typically involve solvent extraction (e.g., hexane or ethyl acetate), followed by chromatographic separation and recrystallization [11].

In this study, we demonstrate for the first time that AA potently inhibits RANKL-induced osteoclast differentiation through pathway-biased suppression of MAPK signaling. This selective mechanism distinguishes AA from conventional anti-resorptive agents.

## 2. Materials and Methods

### 2.1. Reagents and Antibodies

Angelic acid (AA; (≥99% purity, CAS: 565-63-9) was purchased from (HY-N6929. MedChemExpress, Monmouth Junction, NJ, USA). The compound was characterized by the supplier using HPLC and NMR, consistent with established spectroscopic data. Recombinant murine RANKL was from R&D Systems (Minneapolis, MN, USA). Primary antibodies against phospho-ERK1/2 (Thr202/Tyr204) (GB113492), ERK1/2 (ZB12087), phospho-JNK (Thr183/Tyr185) (GB12018), JNK (GB114321), phospho-p38 (Thr180/Tyr182) (GB153380), p38 (GB154685) (all used at 1:1000 dilution) and GAPDH (1:5000 dilution) were procured from Servicebio (Wuhan, Hubei, China).

### 2.2. Cell Culture and Osteoclast Differentiation

RAW264.7 murine macrophage cells were obtained from Wuhan Pricella Biotechnology Co., Ltd. (Wuhan, Hubei, China) and cultured in DMEM containing 10% fetal bovine serum (FBS), 100 U/mL penicillin, and 100 µg/mL streptomycin at 37 °C in a humidified atmosphere with 5% CO_2_. For osteoclast differentiation, cells were stimulated with RANKL (50 ng/mL) in the presence or absence of AA for 5 days. The medium was changed every 2 days. AA was dissolved in DMSO, and the final DMSO concentration in culture media did not exceed 0.1%.

### 2.3. TRAP Staining and Osteoclast Quantification

Cells were fixed with 4% paraformaldehyde and stained using a leukocyte acid phosphatase staining kit (G1050, Servicebio, Wuhan, Hubei, China) according to the manufacturer’s protocol. TRAP-positive multinucleated cells (≥3 nuclei) were counted as osteoclasts. TRAP-positive multinucleated osteoclasts containing three or more nuclei were counted in randomly selected non-overlapping fields captured at 20× magnification. For each condition, at least five fields per well were analyzed and averaged, and osteoclast density was calculated by normalizing counts to the imaged area and expressed as osteoclasts per mm^2^. The osteoclast fusion index was calculated as the percentage of nuclei in multinucleated cells relative to total nuclei. TRAP activity was analyzed using a commercial kit according to the manufacturer’s protocol (EEA055, ThermoFisher Scientific, Waltham, MA, USA).

### 2.4. Cell Viability Assay

Cell viability was measured using Cell Counting Kit-8 (CCK-8) according to the manufacturer’s protocol, Servicebio (Wuhan, Hubei, China). The cells were treated with various concentrations of AA for 1–4 days, and absorbance was measured at 450 nm.

### 2.5. Western Blot Analysis

Cells were lysed in RIPA buffer containing protease and phosphatase inhibitors. Equal amounts of protein (50 µg) were separated by SDS-PAGE, transferred to PVDF membranes, and probed with specific antibodies. Protein bands were visualized using enhanced chemiluminescence and quantified using ImageJ software (Version 1.54p, NIH, Bethesda, MD, USA). 

### 2.6. NF-κB Luciferase Reporter Assay

RAW 264.7 cells stably expressing NF-κB-luciferase reporter (D2206, Beyotime, Shanghai, China) were pre-treated with AA (2 µM) for 1 h, followed by RANKL stimulation for 6 h in the presence or absence of AA. Luciferase activity was measured using the Steady-Glo^®^ Luciferase Assay system from Promega (Madison, WI, USA).

### 2.7. Immunofluorescence Microscopy

Cells were fixed, permeabilized, and immunostained with anti-NFATc1 antibody followed by an Alexa Fluor-conjugated secondary antibody. NFATc1 signals were visualized in red, while nuclei were counterstained with DAPI and pseudocolored in green. Images were captured using a BZ-X810 fluorescence microscope (Keyence Corporation, Osaka, Japan).

### 2.8. Quantitative Real-Time PCR

Total RNA was extracted using a total-RNA isolation Kit (G3640, Servicebio, Wuhan, Hubei, China) according to the manufacturer’s protocol and reverse transcribed into cDNA. qPCR was performed using the SYBR Green master mix on a LightCycler^®^ 96 system (Roche, Basel, Switzerland). Gene expression was normalized to *GAPDH* using the 2^−ΔΔCt^ method. Primer sequences are provided in Supplementary Table S1.

### 2.9. Statistical Analysis

Data are presented as mean ± SEM from at least three independent experiments. Statistical significance between two groups was determined using Student’s *t*-test. For comparisons involving multiple concentrations or multiple time points, one-way or two-way ANOVA with appropriate post hoc testing (Dunnett’s or Tukey’s multiple comparisons test) was applied. All analyses were performed using GraphPad Prism 9.0. *p* < 0.05 was considered statistically significant.

## 3. Results

### 3.1. Angelic Acid Potently Inhibits RANKL-Induced Osteoclast Formation

**Figure 1:**
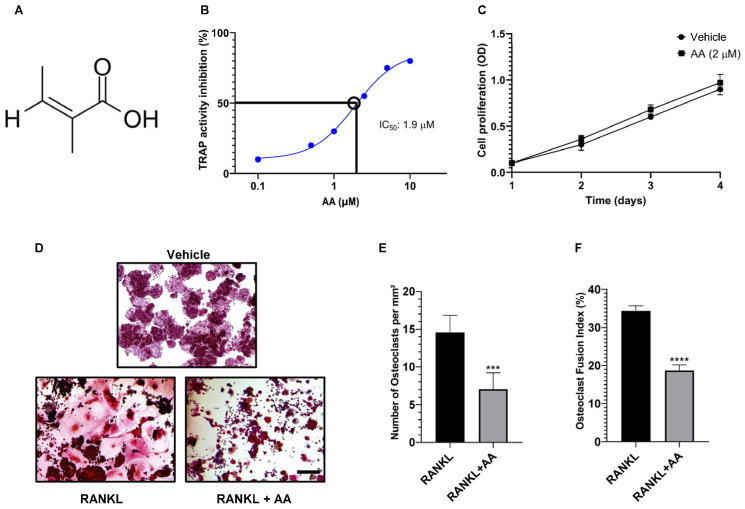
Angelic acid potently inhibits RANKL-induced osteoclast formation without cytotoxicity. (**A**) Chemical structure of angelic acid (AA). (**B**) Dose–response curve showing inhibition of TRAP activity in RAW264.7 cells treated with increasing concentrations of AA in the presence of RANKL. The IC_50_ value was calculated as 1.9 µM. (**C**) Cell viability assessed by CCK-8 assay. RAW264.7 cells were treated with AA (2 µM) for 1–4 days, showing no cytotoxic effects. (**D**) Representative TRAP staining images showing osteoclast formation in vehicle (DMSO), RANKL-treated, and RANKL + AA (2 µM) groups. Scale bar, 50 µm. (**E**) Quantification of TRAP-positive multinucleated cells (≥3 nuclei). (**F**) Osteoclast fusion index calculated as the percentage of nuclei in multinucleated cells relative to total nuclei. Data are presented as mean ± SEM (*n* = 3 independent experiments). *** *p* < 0.001, **** *p* < 0.0001 vs. RANKL-treated control.

To evaluate the effect of angelic acid (AA; Figure 1A) on osteoclast differentiation, RAW264.7 cells were treated with increasing concentrations of AA in the presence of RANKL. TRAP activity analysis demonstrated that AA inhibited RANKL-induced osteoclast differentiation in a dose-dependent manner, with an IC_50_ of 1.9 µM (Figure 1B). CCK-8 assays confirmed that AA at 2 µM did not affect cell viability (Figure 1C), indicating that the observed anti-osteoclastogenic effect was not due to cytotoxicity. Consistently, TRAP staining revealed a marked reduction in multinucleated osteoclast formation in the presence of AA (Figure 1C). Quantitative analysis further demonstrated significant decreases in osteoclast number (Figure 1D) and fusion index (Figure 1E). Together, these results establish AA as a potent inhibitor of RANKL-induced osteoclast differentiation.

### 3.2. Angelic Acid Exhibits Pathway-Biased Suppression of MAPK Signaling

**Figure 2:**
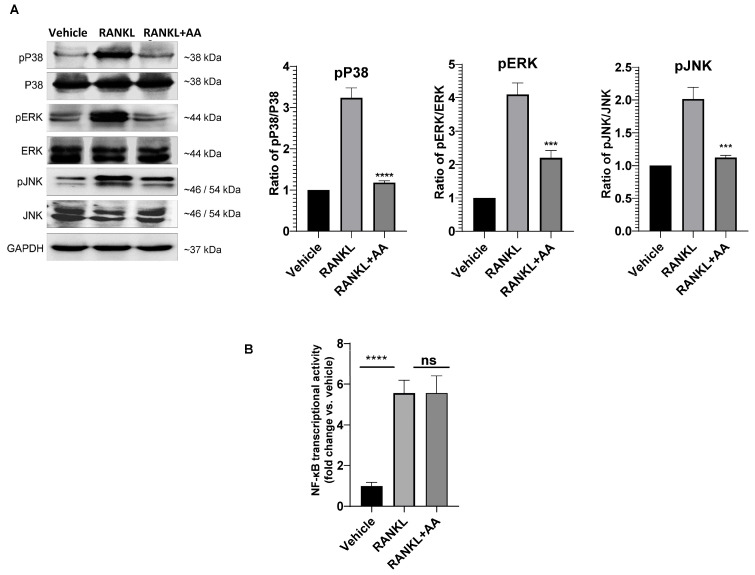
Angelic Acid Exhibits Pathway-Biased Suppression of MAPK Signaling. (**A**) Western blot analysis of MAPK pathway activation. RAW264.7 cells were pretreated with angelic acid (AA; 2 µM) for 1 h followed by RANKL stimulation (50 ng/mL) for 10 min. Cell lysates were analyzed for phosphorylated and total p38, ERK, and JNK. GAPDH served as loading control. Right panels show densitometric quantification of phospho-protein/total protein ratios. Data are mean ± SEM (*n* = 3). *** *p* < 0.001 or **** *p* <0.0001 vs. RANKL-treated control. (**B**) NF-κB transcriptional activity measured by luciferase reporter assay. RAW264.7-NF-κB-luc cells were treated with AA followed by RANKL stimulation. Data are mean ± SEM (*n* = 3). **** *p* <0.0001 vs. vehicle; ns, not significant vs. RANKL.

To elucidate the mechanism, we examined RANKL-activated signaling pathways. Western blot analysis revealed that AA markedly suppressed phosphorylation of all three MAPK family members (p38, ERK, and JNK; Figure 2A), while NF-κB pathway activation assessed by luciferase reporter assay was not significantly affected by AA treatment (Figure 2B). This pathway-biased inhibition distinguishes AA from many natural products, which typically exhibit broader, non-selective modulation of multiple signaling cascades. These data indicate that angelic acid preferentially targets MAPK signaling without suppressing NF-κB transcriptional output.

### 3.3. Angelic Acid Blocks NFATc1 Nuclear Translocation and Osteoclast Gene Expression

**Figure 3:**
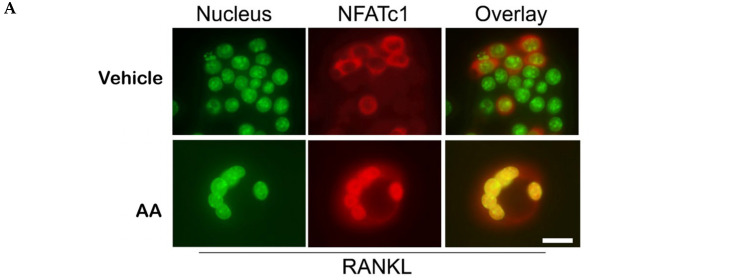

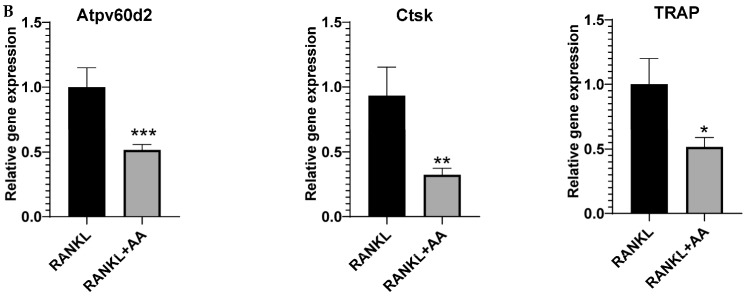
Angelic acid attenuates NFATc1 nuclear translocation and suppresses osteoclast-specific gene expression. (**A**) Immunofluorescence analysis of NFATc1 cellular localization. RAW264.7 cells were treated with RANKL in the presence or absence of angelic acid (AA; 2 µM) for 3 days. Nuclei were stained with DAPI (pseudocolored green), NFATc1 is shown in red, and merged images indicate colocalization (yellow), representing NFATc1 nuclear translocation. Representative images from three independent experiments are shown. Scale bar, 20 µm. (**B**) Quantitative RT-PCR analysis of osteoclast marker genes. RAW264.7 cells were treated with RANKL ± AA (2 µM) for 3 days. Expression of *Atp6v0d2*, *cathepsin K* (Ctsk), and *TRAP* was normalized to *GAPDH*. Data are presented as mean ± SEM (*n* = 3 independent experiments). * *p* < 0.05, ** *p* < 0.01, *** *p* < 0.001 vs. RANKL-treated control.

Given that MAPK and NF-κB signaling converge on NFATc1, the master regulator of osteoclastogenesis [12], we investigated NFATc1 activation. Immunofluorescence microscopy revealed robust nuclear accumulation of NFATc1 in RANKL-stimulated control cells, whereas AA treatment markedly inhibited NFATc1 nuclear translocation (Figure 3A). Consistent with this, quantitative PCR analysis demonstrated that AA significantly reduced expression of *Atp6v0d2*, *cathepsin K*, and *TRAP* (Figure 3B), key mediators of osteoclast function and bone resorption [1].

## 4. Discussion

This study provides the first evidence that angelic acid (AA) potently inhibits osteoclastogenesis through selective suppression of MAPK signaling. The low-micromolar IC_50_ (1.9 µM) indicates greater potency than several reported natural compounds, such as asiatic acid (~10 µM) [13] and andrographolide (~8 µM) [14]. Notably, AA markedly inhibits all three MAPK branches (ERK, p38, JNK) while sparing NF-κB, distinguishing it from most natural products that exhibit non-selective pathway modulation. This pathway-biased profile may reduce potential off-target effects on NF-κB-dependent immune functions [15].

The α,β-unsaturated carboxylic acid structure of AA suggests the potential for covalent modification of nucleophilic residues in upstream signaling proteins via Michael addition [10]. Alternatively, AA might interfere with RANKL-RANK receptor complex formation or TRAF6 recruitment, which are essential for RANKL-induced MAPK activation [16]. Further biochemical studies are needed to identify the direct molecular target(s) of AA. We propose two potential mechanisms of action that warrant future investigation. First, the α,β-unsaturated scaffold of AA may function as a Michael acceptor, forming covalent adducts with cysteine residues in upstream MAPK pathway components, including MAP3Ks or the E3 ubiquitin ligase TRAF6 [10]. Second, AA may interfere sterically or allosterically with RANK–TRAF6 complex assembly, a critical step for MAPK, but not NF-κB transcriptional activation. Validation strategies will include thermal shift assays and covalent capture–mass spectrometry to identify direct binding partners, co-immunoprecipitation to assess TRAF6 recruitment to RANK, and structure–activity relationship studies using AA analogs.

From a translational perspective, AA offers several advantages, including its presence in traditionally used *Angelica* species [7], structural simplicity amenable to analog development, and potential dual benefits in cancer-induced osteolysis given its recently identified ferroptosis-inducing activity [9].

Several limitations should be noted. First, this study focused on in vitro osteoclastogenesis; in vivo efficacy in ovariectomized or inflammatory arthritis models remains to be demonstrated. Second, effects on osteoblasts and bone formation were not examined. Third, while NF-κB transcriptional activity was unaffected, it should be noted that upstream NF-κB signaling events including IκBα degradation and p65 phosphorylation were not evaluated in this study; the observed pathway selectivity therefore specifically refers to the transcriptional output level, and assessment of upstream NF-κB intermediates in future studies would further refine this characterization. Finally, the precise molecular target of AA requires identification through biochemical approaches.

## 5. Conclusions

In conclusion, this study identifies AA as a potent, naturally occurring inhibitor of RANKL-induced osteoclastogenesis. AA acts through preferential suppression of MAPK (ERK1/2, p38, and JNK) signaling, without affecting NF-κB transcriptional activity, thereby blocking NFATc1 nuclear translocation and downstream osteoclast-specific gene expression. The low-micromolar potency, favorable selectivity profile, and structural amenability to analog development make AA an attractive lead compound for therapeutic development in osteoporosis, rheumatoid arthritis, and other osteolytic diseases. Future studies will validate the molecular target(s) and assess in vivo efficacy in established bone-loss models.

## Data Availability

The original contributions presented in the study are included in the article; further inquiries can be directed to the corresponding author.

## Supplementary Materials

The following supporting information can be downloaded at: https://www.mdpi.com/article/10.3390/cimb48040412/s1.

## Abbreviations

AA	Angelic Acid
CCK-8	Cell Counting Kit-8
DMEM	Dulbecco’s Modified Eagle Medium
DMSO	Dimethyl sulfoxide
ERK	Extracellular signal-regulated kinase
FBS	Fetal bovine serum
GAPDH	Glyceraldehyde 3-phosphate dehydrogenase
JNK	c-Jun N-terminal kinase
MAPK	Mitogen-activated protein kinase
NFATc1	Nuclear factor of activated T-cells, cytoplasmic 1
NF-κB	Nuclear factor kappa-light-chain-enhancer of activated B cells
qPCR	Quantitative polymerase chain reaction
RA	Rheumatoid arthritis
RANKL	Receptor activator of nuclear factor-κB ligand
RIPA	Radioimmunoprecipitation assay
RT-qPCR	Reverse transcription quantitative polymerase chain reaction
SDS-PAGE	Sodium dodecyl sulfate polyacrylamide gel electrophoresis
TRAP	Tartrate-resistant acid phosphatase
TRAF6	TNF receptor-associated factor 6:

## References

[1] Boyle W.J., Simonet W.S., Lacey D.L. (2003). Osteoclast differentiation and activation. Nature.

[2] Takegahara N., Kim H., Choi Y. (2024). Unraveling the intricacies of osteoclast differentiation and maturation: Insight into novel therapeutic strategies for bone-destructive diseases. Exp. Mol. Med..

[3] Takayanagi H., Kim S., Koga T., Nishina H., Isshiki M., Yoshida H., Saiura A., Isobe M., Yokochi T., Inoue J.I. (2002). Induction and activation of the transcription factor NFATc1 (NFAT2) integrate RANKL signaling in terminal differentiation of osteoclasts. Dev. Cell.

[4] Sobacchi C., Menale C., Crisafulli L., Ficara F. (2025). Role of RANKL Signaling in Bone Homeostasis. Physiology.

[5] Skjodt M.K., Frost M., Abrahamsen B. (2019). Side effects of drugs for osteoporosis and metastatic bone disease. Br. J. Clin. Pharmacol..

[6] Kaur A., Bhatti R. (2021). Understanding the phytochemistry and molecular insights to the pharmacology of *Angelica archangelica* L. (garden angelica) and its bioactive components. Phytother. Res..

[7] Borlak J., Diener H.C., Kleeberg-Hartmann J., Messlinger K., Silberstein S. (2022). Petasites for Migraine Prevention: New Data on Mode of Action, Pharmacology and Safety. A Narrative Review. Front. Neurol..

[8] Gu D.R., Yang H., Kim S.C., Hwang Y.H., Ha H. (2023). Water Extract of *Angelica dahurica* Inhibits Osteoclast Differentiation and Bone Loss. Int. J. Mol. Sci..

[9] Cao Y., Wang Y., Li Y., Liu S., Wang L., Zhou L., Zhu T. (2025). Angelic acid triggers ferroptosis in colorectal cancer cells via targeting and impairing NRF2 protein stability. J. Nat. Med..

[10] Jackson P.A., Widen J.C., Harki D.A., Brummond K.M. (2017). Covalent Modifiers: A Chemical Perspective on the Reactivity of α,β-Unsaturated Carbonyls with Thiols via Hetero-Michael Addition Reactions. J. Med. Chem..

[11] Sarker S.D. (2012). Natural Products Isolation.

[12] Kim J.H., Kim N. (2014). Regulation of NFATc1 in Osteoclast Differentiation. J. Bone Metab..

[13] Hong G., Zhou L., Han X., Sun P., Chen Z., He W., Tickner J., Chen L., Shi X., Xu J. (2020). Asiatic Acid Inhibits OVX-Induced Osteoporosis and Osteoclastogenesis Via Regulating RANKL-Mediated NF-κb and Nfatc1 Signaling Pathways. Front. Pharmacol..

[14] Zhai Z.J., Li H.W., Liu G.W., Qu X.H., Tian B., Yan W., Lin Z., Tang T.T., Qin A., Dai K.R. (2014). Andrographolide suppresses RANKL-induced osteoclastogenesis in vitro and prevents inflammatory bone loss in vivo. Br. J. Pharmacol..

[15] Liu T., Zhang L., Joo D., Sun S.C. (2017). NF-κB signaling in inflammation. Signal Transduct. Target. Ther..

[16] Deepak V., Yang S.T., Li Z., Li X., Ng A., Xu D., Li Y.P., Oursler M.J., Yang S. (2022). IFT80 negatively regulates osteoclast differentiation via association with Cbl-b to disrupt TRAF6 stabilization and activation. Proc. Natl. Acad. Sci. USA.

