# Supplementary material for: Angelic Acid Prevents RANKL-Induced Osteoclastogenesis Through Pathway-Biased Inhibition of MAPK–NFATc1 Signaling
Source: Curr Issues Mol Biol. 2026 Apr 17;48(4):412. doi: 10.3390/cimb48040412 (PMC13114683; doi:10.3390/cimb48040412)
Supplement: Supplementary file 1 [file cimb-48-00412-s001.zip › cimb-4215769-supplementary.pdf]

Gene	Gene Symbol	NCBI Gene ID	Forward Primer (5′→3′)	Reverse Primer (5′→3′)	Product Length (bp)
Tartrate-resistant acid phosphatase	*Acp5* (TRAP)	11433	CACTCCCACCCTGAGATTTGT	CATCGTCTGCACGGTTCTG	118
Cathepsin K	*Ctsk*	13038	GAAGAAGACTCACCAGAAGCAG	TCCAGGTTATGGGCAGAGATT	102
ATPase H^+ transporting V0 subunit D2	*Atp6v0d2*	242341	CAGAGCTGTACTTCAATGTGGAC	AGGTCTCACACTGCACTAGGT	111
Glyceraldehyde-3-phosphate dehydrogenase	*Gapdh*	14433	AGGTCGGTGTGAACGGATTTG	TGTAGACCATGTAGTTGAGGTCA	123

Supplementary Table S1. Primer sequences used for quantitative real-time PCR analysis. All primers are presented in the 5′–3′ direction.
